# STING agonist diABZI enhances the cytotoxicity of T cell towards cancer cells

**DOI:** 10.1038/s41419-024-06638-1

**Published:** 2024-04-13

**Authors:** Ling Wang, Zhaoduan Liang, Yunzhuo Guo, Jean de Dieu Habimana, Yuefei Ren, Obed Boadi Amissah, Omar Mukama, Siqi Peng, Xuanyan Ding, Linshuang Lv, Junyi Li, Min Chen, Zhaoming Liu, Rongqi Huang, Yinchao Zhang, Yi Li, Zhiyuan Li, Yirong Sun

**Affiliations:** 1 Division of Life Sciences and Medicine, University of Science and Technology of China, Hefei, 230026 China; 2 CAS Key Laboratory of Regenerative Biology, Guangdong Provincial Key Laboratory of Stem Cell and Regenerative Medicine, Guangzhou Institutes of Biomedicine and Health, Chinese Academy of Sciences, Guangzhou, 510530 China; 3 Bioland Laboratory (Guangzhou Regenerative Medicine and Health GuangDong Laboratory), Guangzhou, 510005 China; 4 Department of Biology, College of Science and Technology, University of Rwanda, Kigali, 3900 Rwanda; 5 State Key Laboratory of Respiratory Disease, Guangzhou Institutes of Biomedicine and Health, Chinese Academy of Sciences, Guangzhou, Guangdong China; 6 Department of Breast Surgery, Second Hospital of Jilin University, Changchun, 130022 China; 7 GZMU-GIBH Joint School of Life Sciences, Guangzhou Medical University, Guangzhou, 510530 China

**Keywords:** Cancer immunotherapy, Immune cell death

## Abstract

Antigen-specific T cell receptor-engineered T cell (TCR-T) based immunotherapy has proven to be an effective method to combat cancer. In recent years, cross-talk between the innate and adaptive immune systems may be requisite to optimize sustained antigen-specific immunity, and the stimulator of interferon genes (STING) is a promising therapeutic target for cancer immunotherapy. The level of expression or presentation of antigen in tumor cells affects the recognition and killing of tumor cells by TCR-T. This study aimed at investigating the potential of innate immune stimulation of T cells and engineered T cells to enhance immunotherapy for low-expression antigen cancer cells. We systematically investigated the function and mechanism of cross-talk between STING agonist diABZI and adaptive immune systems. We established NY-ESO-1 full knockout Mel526 cells for this research and found that diABZI activated STING media and TCR signaling pathways. In addition, the results of flow cytometry showed that antigens presentation from cancer cells induced by STING agonist diABZI also improved the affinity of TCR-T cells function against tumor cells in vitro and in vivo. Our findings revealed that diABZI enhanced the immunotherapy efficacy of TCR-T by activating STING media and TCR signaling pathways, improving interferon-γ expression, and increasing antigens presentation of tumor cells. This indicates that STING agonist could be used as a strategy to promote TCR-T cancer immunotherapy.

## Introduction

Immunotherapy is a groundbreaking technique in the realm of cancer treatment; however, there is a subset of patients who do not exhibit a response to this type of therapy. It is encumbered by tumor heterogeneity, loss of tumor-specific antigen targets, and the regulatory tumor environment [1]. T cell-based immunotherapy has proven to be an effective method to combat cancer. T cell receptor-engineered T cell (TCR-T) and chimeric antigen receptor T cell (CAR-T) are adoptive T cell-based therapies that genetically modify natural T cells for cancer immunotherapy. CAR-T therapy yields significant efficacy in B-cell hematological malignancies, whereas the bulk of solid tumors are less responsive to CAR-T cells [2]. TCR-T therapy is drawing more attention due to its unprecedented prospect in the treatment of solid tumors [3].

Peptides derived from disease-associated proteins can be presented on the cell surface as peptide–human leukocyte antigen (pHLA) complexes [4]. The antitumor response is driven by the T cell receptor (TCR) which can specifically recognize the pHLA presented from the tumor cells. Antigen-specific T cell therapy has shown a good efficiency against melanoma. NY-ESO-1 is the most targeted antigen type in many cancers [5]. T cells expressing NY-ESO-1-specific high-affinity T cell receptors (HATs) have provided effective antitumor responses in clinical trials of melanoma, myeloma, and synovial cell sarcoma [6]. However, the expression level of NY-ESO-1 varies in different cancer cells. For example, despite the fact that most melanoma cell lines express NY-ESO-1, HLA-A*0201-positive Mel526 shows a significantly lower expression level of NY-ESO-1 [7] compared to others. The level of expression or antigen presentation in tumor cells affects the recognition and killing of tumor cells by HATs. As such, the improvement of antigen presentation and T cell killing efficiency in tumor microenvironment is receiving more attention in immunotherapy.

Novel strategies to target innate immunity in cancer therapy have emerged in recent years, including the DNA sensing cGAS/STING pathway [8, 9], Toll-like receptors (TLRs) [10], nucleotide-binding oligomerization domain-like receptors NLRP3, and the retinoic acid-inducible gene-I (RIG-I)-like receptors (RLRs) [11, 12]. STING agonists DMXAA and cGAMP greatly enhanced CAR-T cell persistence in the tumor microenvironment as reported recently [13]. The Cross-talk between the innate and adaptive immune systems may be requisite to optimize sustained antigen-specific immunity [14].

The STING pathway is an innate immune pathway that activates several downstream signaling events, the most commonly known being IRF3 activation and *IFN-β* gene expression [15]. STING is a novel potential target, and STING agonists have recently shown potential application in cancer immunotherapy [16]. Therefore, STING agonists are being developed as a novel cancer therapeutic, and a better understanding of STING pathway regulation is leading to a broadened list of candidate immune regulatory targets [17]. The STING protein canonically senses cyclic dinucleotides and induces the expression of type I IFN and pro-inflammatory cytokines [18]. The type I IFN signaling plays a crucial role in innate immune responses and exerts a variety of effects on different immune cells, including enhancing the cytotoxic ability of natural killer cells and their potential to secrete IFN-γ [19]. STING agonist diABZI was recently reported to potentiate antitumor immunity and radiotherapy for glioblastoma [20].

This study aimed at investigating the potential of innate immune stimulation of T cells and engineered T cells to enhance immunotherapy for low-expression antigen cancer cells. Specifically, the research examined the efficiency and mechanism of cross-talk between STING agonist diABZI and TCR-T when this combined approach were applied to Mel526 tumor cells in vitro and in vivo.

## Materials and methods

### Mice and mice experiment

Immunodeficient NCG mice (NOD/ShiLtJGpt- *Prkdc*^*em26Cd52*^*Il2rg*^*em26Cd22*^/ Gpt) aged 6 weeks were purchased from GemPharmatech CO., Ltd (JS, China). Experimental protocols were approved by the Institutional Animal Care and Use Committee of Guangzhou Institutes of Biomedicine and Health, CAS (IACUC 2024002). Each mouse was injected subcutaneous of armpit with 1.5 × 10^6^ Mel526 cells at first two days. After the tumors reached nearly 60 mm^3^ (day 12), the male mice were randomly assigned to five groups and treated with phosphate-buffered solution (PBS), 3.0 × 10^6^ TCR-T cells, 0.1 μM diABZI, 3.0 × 10^6^ diABZI pro-treated 2 h (hours) TCR-T cells or 3.0 × 10^6^ diABZI pro-treated 2 h TCR-T cells plus 0.1 μM diABZI. TCR-T cells via tail vein injection and diABZI injected to tumors. Tumor volumes were measured every 2 days (V = π/6 × L × W × H, where V is tumor volume, L is tumor length, W is tumor width, and H is tumor height). According to IACUC standards, the maximum allowable size for subcutaneous tumors in mice is 20 mm in diameter. So, euthanasia was performed on tumor-bearing mice, and tumor tissue was removed and weighed on the 22nd day.

### Cells and stimulated molecules

The melanoma cell line, Mel526, and breast carcinoma cell line, MDA-MB-231, were kindly provided by Xiangxue Life Sciences Research Center (Guangzhou, China). *NY-ESO-1*^*−/−*^ Mel526 was constructed by CRISPR/Cas9 system, and NY-ESO-1 CRISPR/Cas9 KO plasmids were purchased from Santa Cruz Biotechnology (SC-418340, Dallas, USA). The lentivirus packaging cell line HEK-293T (RRID: CVCL_0063) was purchased from the ATCC (Rockville, USA) and cultured in DMEM containing 10% FBS.

Peripheral blood mononuclear cells (PBMC) were isolated from the peripheral blood samples of healthy volunteers by density gradient centrifugation using Ficoll-Isopaque (Axis shield) which reported before [21]. An informed consent was obtained from the volunteers who donated blood samples. PBMCs were used to make peripheral blood lymphocytes (PBLs), which were then stimulated with Human T-Activator CD3/CD28 Dynabeads (Life Technologies, Carlsba, USA) in RPMI 1640 medium with 10% fetal bovine serum and 100 IU of recombinant human IL-2 [22]. STING inhibitor and agonist, H151 and diABZI, were purchased from InvivoGen (Hong Kong, China). T cells and tumor cells were treated for 3 h separately, completely washed off with PBS twice, and then co-cultured.

### Construction of TCR-T

A lentiviral vector was produced at the City of Hope (Duarte, CA, USA) using transient transfection with four plasmids expressing the transfer vector, Rev, VSV-G, and gag/pol, in 293T cells. The TCR gene constructs were subcloned into a third-generation lentiviral vector. WT-TCR was cloned with the CDR3β amino acid sequence, GLAGGRPEQYF, and 1G4 TCR with the variable and junctional segment, SLLMWITQC, which originated from NY-ESO-1 as previously reported [5]. The supernatant was collected at multiple time points, clarified, treated with benzonase, and concentrated by tangential flow filtration and centrifugation. The transduction efficiency was measured with primary T cells. Lentiviral particles were generated by transfecting HEK-293T cells and stored at −80 °C.

Engineered T cells were generated from CD4^+^ and CD8^+^ T cells that were activated, expanded using anti-CD3/28 antibody conjugated paramagnetic microbeads (Life Technologies, Carlsbad, CA), and transduced with TCR packaged lentivirus at a multiplicity of infection of one transducing unit (TU)/cell as previously described [22]. After 72 h, TCR expression was assessed by flow cytometry and the cells were expanded for further 7–10 days for complete release testing.

1G4 TCR was combined with HLA-A2 restrictive NY-ESO-1 complex [23]. The 1G4 TCR has an affinity of approximately 32 μM (equilibrium dissociation constant K_d_) for wild-type HLA-A2-NY-ESO, while a higher affinity version (1G4 HA) was selected using phage display technology following a previously reported method [5]. In this study, two versions of the TCR, namely 1G4 WT-T (with an affinity of 32 μM) and 1G4 HA-T (with an affinity of 1.07 μM) were used.

### Cytotoxicity assay

Lactate dehydrogenase (LDH) released during cell lysis was measured using a CytoTox 96 Non-Radioactive Cytotoxicity Assay Kit (Promega, cat. No. G1780, Madison, USA) according to the manufacturer’s instructions. Effector and target cells Mel526 with or without stimulated molecules were co-cultured at various ratios for 24 h, and the percentage of specific killing TCR-T was calculated using the following formula: % Cytotoxicity = [(Experimental release - Effector spontaneous release - Target spontaneous release)/ (Target maximum release - Target spontaneous release)] ×100. The cytotoxicity analyses were repeated in three independent experiments with biological replicates.

### Flow cytometry

APC-conjugated anti-human TCRα/β (Biolegend cat. No. 317308, CA, USA) and PE fluorescent-labeled anti-human CD3 (Biolegend cat. No. 306717, CA, USA) were used to detect T cells with transduced TCR. Cells were stained for 30 min at room temperature. Flow cytometry analysis was performed by Guava easyCyte flow cytometer (Millipore, MA, USA). PE-conjugated anti-HLA-A2 (ARG5395, Arigobio, China) were used to detect HLA of tumor cells. Two or three independent experiments are performed. Cells obtained from tumor tissues by Tumor Dissociation Kit follow the manufacturer’s protocol (Miltenyi Biotec, CA, USA).

### Western blot analysis

Cells were lysed with NP 40 buffer containing a protease inhibitor (Cocktail, CAS), and protein from tumor were extracted by a Tissue homogenizer. Total protein, 20 μg, was separated on a 12% SDS-PAGE, transferred to a PVDF membrane, and blocked with TBS-buffer containing 5% skim milk and 0.05% Tween-20. The membrane was overlaid with a 1:1000 dilution primary antibody which was purchased from Cell Signaling Technology in blocking buffer. The membrane was washed three times with TBS-Tween (Solarbio.Co., Beijing, China) and overlaid with a 1:2000 dilution of anti-rabbit or anti-mouse antibodies conjugated with alkaline phosphatase (Biosource, MO, USA). After the membrane was washed, staining was performed as described previously [24, 25]. Blot is representative with three independent experiments.

### Antibodies

Antibodies used in this study, Phospho-p38 (Thr180/Tyr182), Phospho-STING (Ser366) (E9A9K), Phospho-Zap-70 (Tyr493)/ Syk (Tyr526), Phospho-NF-κB p65 (Ser536) (93H1), Phospho-IRF3 (Ser396) (D6O1M), Phospho-TBK1/NAK (Ser172) (D52C2), STING (D2P2F), Zap-70 (D1C10E), NF-κB p65 (L8F6), IRF3 (D6I4C), TBK1/NAK (D1B4), PARP (46D11), Phospho-Histone H2A.X (Ser139) (D7T2V), Cleaved PARP (Asp214) (D64E10), Caspase 3 (9662), β-Actin (13E5), Cleaved caspase 3 (Asp175) (5A1E), Stat1 (D1K9Y), Phospho-Stat1 (Tyr701) (D4A7), were purchased from Cell Signaling Technology (Massachusetts, USA). The NY-ESO-1 Polyclonal antibody was purchased from Proteintech (Rosemont, IL, USA).

### RNA extraction and qPCR analysis

The cells and tumors from mice with and without treatments were collected, and the total RNA was isolated using trizol reagent (Invitrogen, Massachusetts, USA) following the manufacturer’s protocol. The concentration and purity of RNA were determined using the GeneQuant RNA/DNA Calculator (Pharmacia-Biotech, Cambridge, UK) and reverse-transcribed using the HiScript II Q RT SuperMix (Vazyme, Nanjing, China). qRT-PCR was performed by using specific primers in a CFX96 Real-Time System (Bio-Rad, USA). The primer sequences for qRT-PCR were obtained from IGEbio (Guangzhou, China) and are listed in Supplementary Table 1. The qPCR analyses were repeated in two independent experiments using three biological replicates as previously described [26]. Differentially expressed genes were identified using a cutoff of fold change >2 and a *p* value < 0.01.

### Cell immunofluorescence

The cells in the culture glasses were fixed in 4% paraformaldehyde for 15 min and permeabilized with 0.2% Triton X-100 and 10% bovine serum albumin (BSA) for 45 min. Next, the cells were incubated with specific primary antibodies overnight at 4 °C and then incubated with secondary antibodies in 10% BSA containing 0.1% Triton X-100. Nuclei were counterstained with DAPI for 5 min. Images were acquired with a fluorescence microscope (Olympus, IX73, and Japan). Two independent experiments are performed. The primary anti-NY-ESO-1 markers were from Cell Signaling Technology, and the secondary antibody with fluorescence Alex Fluor™ 488 and Alex Fluor™ 647 were from Invitrogen (Massachusetts, USA).

### Tumor tissue immunofluorescence and immunohistochemistry

Histological melanoma tumor sections were stained with hematoxylin and eosin stains (H&E) or antibodies for immunofluorescence (IF) and immunohistochemistry (IHC). Primary anti-human cleaved caspase 3 (Cell Signaling Technology) monoclonal antibody was used for IHC. Alex Fluor™ 647 anti-human NY-ESO-1 (BioLegend, CA, USA) and FITC anti-human HLA-A2 (BioLegend, CA, USA) were used for IF. The sections were imaged with a TissureFAXS PLUS, and images were analyzed with TissueFAXSViewer.

### Enzyme-linked immunosorbent assay

Tumor samples were homogenized and levels of IL6 were measured and quantified using enzyme-linked immunosorbent assay (ELISA). IL6 produced in tumors were detected by Quantikine® ELISA Kit (R&D Systems, USA). Two independent experiments with biological replicates were conducted. All protocols were conducted according to the manufacturer’s instructions.

### Statistics

Data were reported as mean ± standard deviation (s.d), and error in figures represent s.d of technical replicates. The statistical significance of the mean differences was determined by using an unpaired two-tailed Student’s *t*-test. A value of *p* < 0.05 was considered statistically significant. A one-way ANOVA (Analysis of Variance) followed by Bonferroni’s post hoc test or F-test was used to determine significant differences (*p* < 0.05) between groups.

## Results

### diABZI induces the cytotoxicity of T cells containing TCR with enhanced affinity by affinity-improved-engineering

**Fig. 1:**
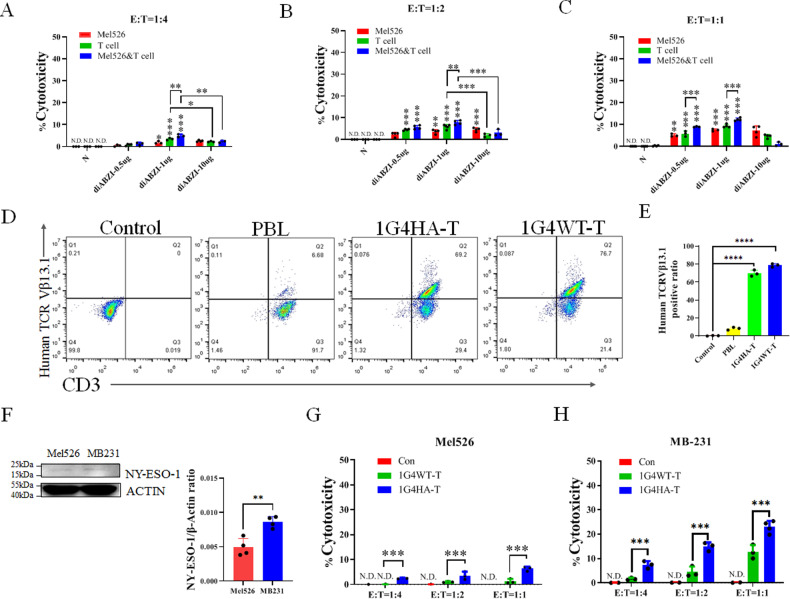
Activation of STING and high-affinity TCR increases T cell cytotoxicity against tumor cells. **A**–**C** Specific lysis by T cells, Mel526, or both cells (Mel526&T cell) treated with diABZI. T cells or Mel526 cells were treated with diABZI as indicated for 3 h separately, then co-cultured at indicated ratios. **D**, **E** Analysis of lentivirus transfection in T cells. T cells were detected by FITC anti-Human TCR Vβ13.1 and APC anti-Human CD3. **F** NY-ESO-1 expression in Mel526 and MB-231. **G**, **H** Cytotoxicity of T cell receptor-engineered 1G4 WT-T cells and 1G4 HATs to Mel526 (**G**) and MB-231 (**H**). T, target cell Mel526, E, effector cells, N.D., not detected. The specific lysis was measured as described in “Methods” section. *p < 0.05, **p < 0.01, ***p < 0.001.

T cells were prepared from PBMC and administered with the STING agonist diABZI and the STING inhibitor H151 to examine the cross-talk between STING and T cells (Fig. 1A–C and Fig. S1A). Our findings revealed that the agonist diABZI significantly increased T cell toxicity towards tumor cells Mel526 (Fig. 1A-C), while the inhibitor H151 did not have any considerable effect (Fig. S1A). The T cell toxicity against tumor cells was enhanced by increasing the reagent concentration from 0.5 to 1 μg/ml, but the higher concentration (10 μg/ml) of diABZI abated the cytotoxicity (Fig. 1A–C). The treatment of tumor cells with the STING agonist diABZI alone showed little lysis activity and increased by the number of T cells and concentrations of diABZI (Fig. 1A–C). A mild lysis activity was detected when the number of effector cells (E) was increased to match the level of target cells (T), as shown in Fig. 1A–C. Additionally, the results indicated a substantial rise in specific lysis when both the target cells, Mel526, and T cells, were treated with diABZI, in contrast to T cells that were treated with diABZI alone (Fig. 1C).

The TCR-T cells were carried as previously reported [5]. The results showed that 70–80% of T cells were transfected with a lentivirus containing engineered TCR, 1G4 HA (Fig. 1D, E) and the total TCR increased to about 20% in these engineered T cells (Fig. S1B, C). The 1G4 HA-T cells exhibited strong cytotoxicity to tumor cells, as measured by assessing the lysis of Mel526 and MB-231, which was higher than WT-TCR transfected lentivirus (Fig. 1G, H). The activity of cytotoxic T lymphocytes (CTLs) was increased with the increase in number of TCR-T cells (Fig. 1G, H). The NY-ESO-1 was about 2-folds higher in MB-231 compared to Mel526 (Fig. 1F). As NY-ESO-1 is the target of 1G4 TCR-T, our results showed that 1G4 TCR-T had higher cytotoxicity towards target cells with high expression of NY-ESO-1(Fig. 1G, H). The T cells with high-affinity TCR substantially killed higher antigen expression tumor cells MB-231, which were about 3-folds higher than low antigen expression Mel526 cells.

### diABZI enhanced the cytotoxicity of HATs

**Fig. 2:**
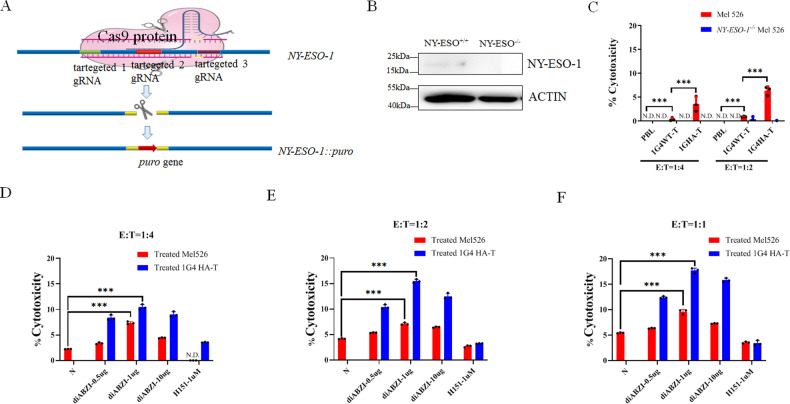
diABZI increased CTLs of TCR-T. **A** The Cas9 knockout of *NY-ESO-1* in Mel526 cells. **B** Western blot of NY-ESO-1 expression in Mel526 cells and knockout cells. **C** The 1G4 HA TCR-T increased lysis in Mel526 cells but not in *NY-ESO-1*^*−/−*^ Mel526 cells. **D**–**F** diABZI increased cytotoxicity of 1G4 TCR-T activity to Mel526 cells at indicated ratios. The specific lysis of diABZI treated with 1G4 TCR-T and Mel526 as indicated. N, no treatment. N.D., not detected. ***p < 0.001.

To investigate whether the STING agonists could increase cytotoxicity of TCR-T activity to tumor cells with low antigen expression, we knocked out the NY-ESO-1 gene in Mel526 cells since different tumor cells have different genetic backgrounds. The WB results showed that no NY-ESO-1 was expressed in *NY-ESO-1*^−/−^ Mel526 cells in which NY-ESO-1 gene was knocked out (Fig. 2A, B), whereas Mel526 cells had low NY-ESO-1 expression, as previously reported [7] (Fig. 2B). We further investigated the cytotoxicity of 1G4 TCR-T activity to *NY-ESO-1*^−/−^ Mel526 and Mel526, and the results indicated that the NY-ESO-1 knockout reduced the toxicity of 1G4 WT-T and HAT cells towards the tumors due to the absence of NY-ESO-1 (Fig. 2C). Furthermore, diABZI-treated 1G4 TCR-T cells exhibited over two folds higher cytotoxicity compared to untreated cells (Fig. 2D–F). Besides, diABZI-treated tumor cells showed a significant increase in LDH release compared to tumor cells without diABZI treatment (Fig. 2D–F). The different concentrations of diABZI and T cell numbers had different effect on tumor cells as shown in Fig. 2D, E, and T cells with a concentration of 1 μg/ml had the best cytotoxicity effect.

These results indicate that STING agonist diABZI significantly enhances T cells and HA TCR-T cytotoxicity against tumors.

### diABZI activated STING-mediated canonical signaling pathway

**Fig. 3:**
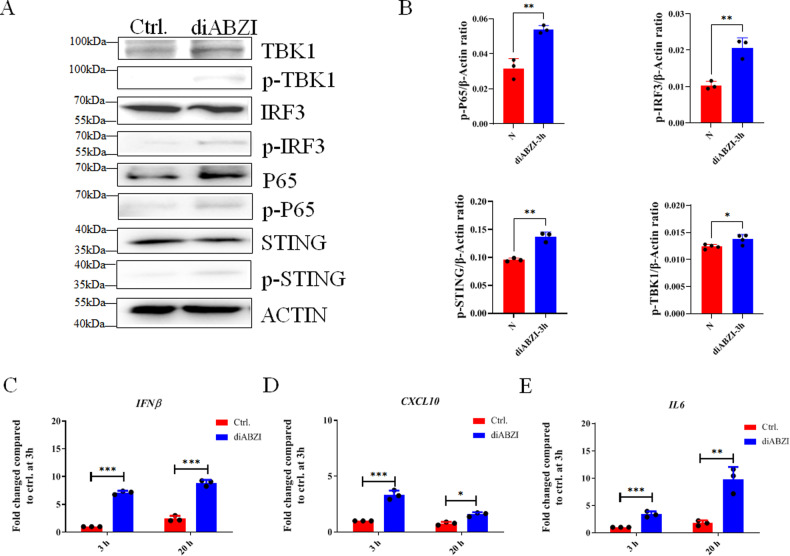
diABZI stimulated classical STING-mediated signaling pathway. **A** Phosphorylation of STING, TBK1, IRF3, and P65 in the STING pathway after diABZI stimulation for 3 h in HATs. **B** Analysis of phosphorylation of ZAP70 and P38. **C**–**E** Gene expression of IFN-β (**C**), CXCL10 (**D**), and IL6 (**E**) in HATs at the time indicated after diABZI stimulation for 3 h. The cells were stimulated by diABZI for 3 h, washed once, and incubated in fresh media for a total of 20 h. N, without diABZI treatment. *p < 0.05, **p < 0.01, ***p < 0.001.

The activation of STING pathway leads to the activation of multiple signaling cascades, thereby resulting in the phosphorylation of IRF3, NF-κB, and TBK1. The interferon regulatory factor 3 (IRF3) is responsible for the induction of type I interferons, while the NF-κB pathway induces pro-inflammatory cytokines. In 1G4 HA-T cells, diABZI stimulation resulted in increased phosphorylation of STING after 3 h, and STING-mediated signal pathway activated at 3 h after stimulation (Fig. 3A, B). All the proteins, IRF3, NF-κB, and TBK1, were also phosphorylated at 3 h after stimulation (Fig. 3A, B).

Moreover, the diABZI stimulation significantly increased the expression of type I interferon IFN-β in HATs and PBLs (Fig. 3C and Fig. S2A, B), as well as the chemical cytokine CXCL10 at 3 h, which decreased after 20 h but still remained higher than that of untreated samples (Fig. 3D and Fig. S2C). The pro-inflammatory cytokine IL6 was also significantly increased after 3 and 20 h of diABZI stimulation (Fig. 3E and Fig. S2D). Therefore, these findings suggest that diABZI activates the canonical STING pathway and increases the expression of CXCL10, IL6, and type I interferon IFN-β at short time.

### diABZI enhanced TCR signaling

**Fig. 4:**
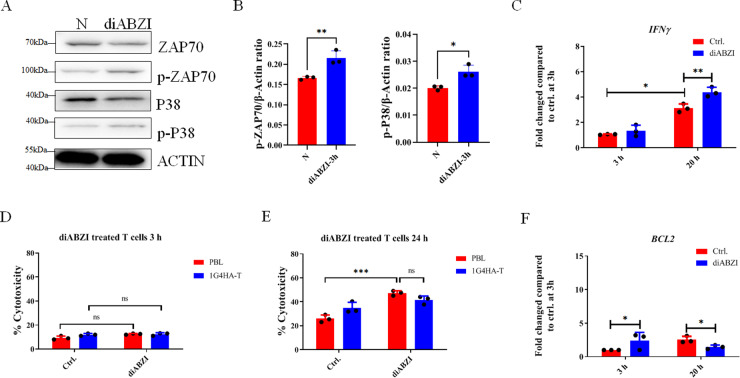
diABZI increased the activation of TCR signaling. **A** TCR signaling pathway identified by western blotting. HATs were treated with diABZI for 3 h. **B** Analysis of phosphorylation of ZAP70 and P38. **C** The expression of IFN-γ measured by RT-qPCR. **D**, **E** Cytotoxicity of T cells treated with diABZI for 3 h (**D**) and 24 h (**E**). The cells were stimulated by diABZI for 3 h, washed once, and incubated in fresh media for a total of 24 h. **F** BCL2 expression in TCR-T cells at the time indicated after treatment with diABZI for 3 h. N, without diABZI treatment. Ctrl., without diABZI treatment. ns, not significant. *p < 0.05, **p < 0.01, ***p < 0.001.

The TCR signaling in HATs was investigated with or without diABZI. The results showed that diABZI treatment for 3 h enhanced TCR signaling (Fig. 4). The activation of STING by diABZI resulted in significant increase of ZAP70 phosphorylation (Fig. 4A, B) and further induced the phosphorylation of P38 (Fig. 4A, B). This indicates that TCR signaling was upgraded by diABZI stimulation. The release of IFN-γ from T cells induced tumor cell death, and their expression increased in HATs after culture with tumor cell Mel526 (Fig. 4C). Moreover, the treatment of HATs cells (Fig. 4C) and PBLs (Fig. S3A) with diABZI increased IFN-γ expression after 20 h. This indicates that diABZI increases T cell cytotoxicity by enhancing TCR signaling and increasing in IFN-γ expression at long time.

The toxicity of diABZI towards T cells was detected by LDH after treating the cells for 3 h. The cell cytotoxicity was not significantly different between samples with and without diABZI; however, a significant increase in cell cytotoxicity was observed after 24 h (Fig. 4D, E). We further found that the expression of BCL2, which encodes an anti-apoptosis protein, was induced in HATs and PBL after diABZI treatment for 3 h (Fig. 4F and Fig. S3B), but its expression had a little decrease after 20 h. BH3 molecules, BIM, BAD, and PUMA were not affected after 3 h of treatment with diABZI at low does, but NOXA had some effect (Fig. S3C–F). But the high doses of diABZI have some effects on increasing BIM, PUMA expression (Fig. S3C, E). This indicates that the treatment of HATs with diABZI for a short time do not increase their cell death.

### diABZI enhanced NY-ESO-1 presentation in tumor cells and tumor cell apoptosis at long time

**Fig. 5:**
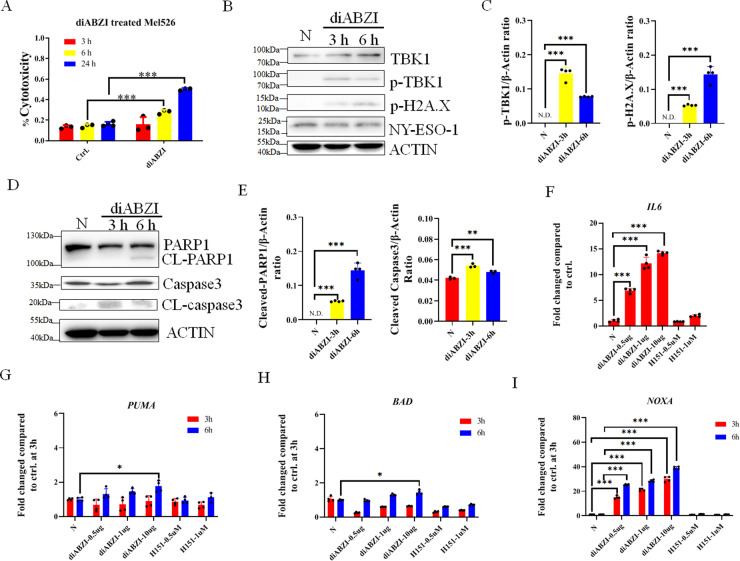
diABZI increased tumor apoptosis at 6 h. **A** diABZI increased the cytotoxicity at different time points indicated in Mel526 cells after diABZI treatment for 3 h. **B**, **C** The phosphorylation of TBK1 and H2Ax increased after diABZI treatment at indicated times. **D**, **E** The diABZI treatment enhanced the cleavage of PARP1 and Caspase 3. **F** Expression of *IL6* at the time indicated after diABZI stimulation. **G**–**I** Expression of BH3 molecules at the time indicated after diABZI stimulation. *p < 0.05, **p < 0.01, ***p < 0.001.

We found that diABZI significantly promoted the death of Mel526 cells after 6 h and 24 h of stimulation, but there was no significant difference within 3 h (Fig. 5A). By studying STING-mediated signaling pathways, we found that diABZI induced phosphorylation of TBK1, IRF3 and H2A.X, but H151 showed no effect on these proteins (Fig. 5B, C, Fig. S4A). The hyper phosphorylation of P65 was found in Mel526 with and without stimulation, which was in accordance with a previously reported study [27] (Fig. S4A). We further found that diABZI promoted the cleavage of apoptosis markers (PARP1 and Caspase-3) after 6 h (Fig. 5D, E).

The apoptosis regulators, BCL2 and BCL-xL, are critical anti-apoptotic proteins [28, 29]. The expression of BCL2 and BCL-xL was not affected by diABZI, but a concentration of 1 μM H151 had some effect after 3 h of treatment (Fig. S4B, C). IL6 significantly increased after 3 h of diABZI treatment (Fig. 5F). The treatment of diABZI for 6 h but not 3 h induced the expression of BH3 molecular BAD and PUMA (Fig. 5G, H), however, NOXA expression was significantly enhanced from 3 h onwards (Fig. 5I). Notably, various genes were significantly attenuated as a result of STING inhibition (Fig. 5G–I).

**Fig. 6:**
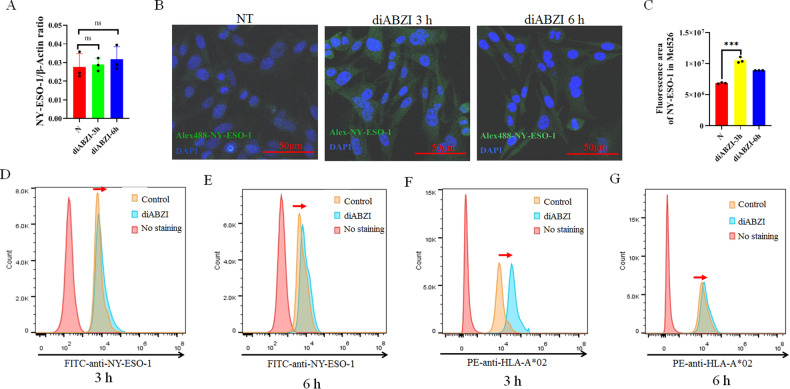
Analysis of antigen presentation in Mel526 cells. **A** Expression of NY-ESO-1 in Mel526 with and without diABZI treatment. **B** Immunofluorescence imaging of NY-ESO-1 expression with and without diABZI treatment. **C** Analysis of imaging results of NY-ESO-1 in cells after diABZI treatment. N, without diABZI treatment. **D**–**G** Mel526 cells were detected by FITC anti-Human NY-ESO-1 (**D**, **E**) and PE anti-Human HLA-A2 (**F**, **G**) after treatment with diABZI for 3 h (**D**, **F**) and 6 h (**E**, **G**). ns, not significant. ***p < 0.001.

The expression of NY-ESO-1 was not changed with or without diABZI (Fig. 5B, Fig. 6A). The Results from immunofluorescence showed that diABZI increased NY-ESO-1 diffusion in Mel526 cells (Fig. 6B, C). It may be because the diABZI enhances NY-ESO-1 presentation in Mel526 cells, which is recognized easily by the antibody. We further detect the presentation of HLA-A2 and NY-ESO-1 in Mel526 cells by flow cytometer with and without diABZI treatment. As shown in Fig. 6D, E, diABZI caused NY-ESO-1 to move to the right side, which implies that the presentation of NY-ESO-1 in Mel526 cells increased after diABZI treatment for 3 h and 6 h. Interestingly, the HLA-A2 presentation on the surface of target cells was also increased by diABZI stimulation, especially after 3 h of treatment (Fig. 6F, G). However, the STING inhibitor H151 did not increase the NY-ESO-1 presentation of Mel526 cells even after 6 h of treatment (Fig. S4D). This suggests that diABZI enhances the killing of tumor cells by TCR-T’s through increasing the presentation of NY-ESO-1.

These results indicated that diABZI enhanced the major histocompatibility complex (MHC) presentation in tumor cells. It can also explain why stimulating tumor cells by STING agonist can increase the cytotoxicity of T cells.

### STING agonist significantly enhance TCR-T cells toxicity to Mel526 tumors in NCG mice

**Fig. 7:**
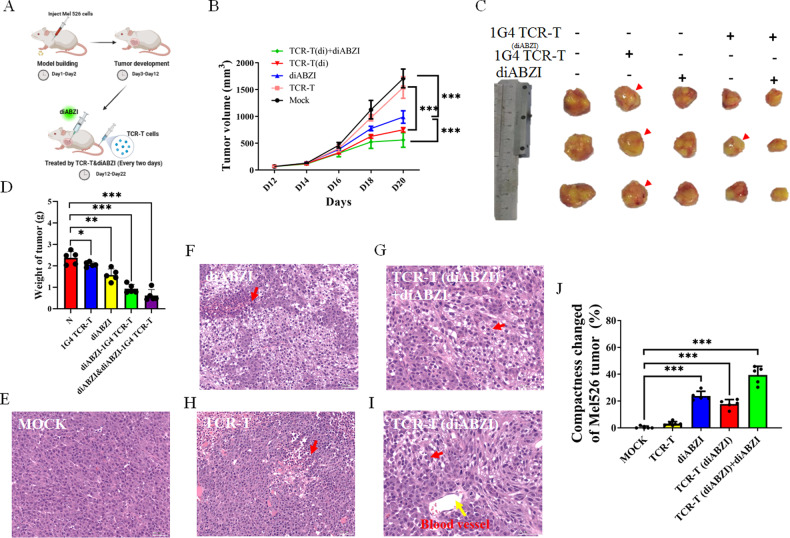
STING agonist diABZI impairs tumor growth and induces tumor regression in combination with TCR-T cells immutherapy. **A** Schematic diagram showing the experiments performed in mice created with BioRender.com. **B** TCR-T cells or diABZI therapy is administered every 2 days via tail vein injection or intratumoral injection, starting from the 12th day after subcutaneous injection of Mel526 cells into NCG mice, Tumor volume (mm^3^) was measured as indicated. **C**, **D** The volumes (**C**) Representative tumors in each group are demonstrated and weights (**D**) of tumors in different groups (*n* = 5) after treatments of 10 days as indicated. Tumor volume and weight were calculated as indicated in Materials and methods. **E**–**I** Photomicrographs of H&E stains of human Mel526 tumors isolated from NCG mice after treatments. **J** Reduced density of tumor tissue after treatment. The relative areas of different groups were calculated. TCR-T (diABZI), TCR-T cells were pre-treated with 1 μg/ml 2 h. *p < 0.05, **p < 0.01, ***p < 0.001.

To further determine the cross-talk between STING agonist and TCR-T cells in vivo, we evaluated the antitumor effects of diABZI and 1G4 TCR-T cells in a Mel526 tumor-bearing NCG mouse model (Fig. 7A). Notably, individual TCR-T cells or diABZI showed few or moderate inhibition on tumor growth as compared to the control groups, while diABZI pre-treated TCR-T cells or combination diABZI significantly suppressed the tumor growth after 8 days, especially combination therapy stopped tumor growth after 6 days (Fig. 7B). The tumor size in the combination treatment group was significantly smaller than that in the other groups, and the antitumor effect of diABZI pre-treatment TCR-T cells were also stronger than that of the individual treatment groups (Fig. 7B–D).

We found that diABZI can also significantly reduce tumor size directly (Fig. 7B–D). Although 1G4 TCR-T cells have a weak antitumor effects due to low expression of target antigens in Mel526 tumors, TCR-T cells does indeed alter the morphology of Mel526 tumors. Compared with untreated ones, TCR-T cells treatment resulted in smoother surface (Fig. 7C).

The Mel526 tumor area exhibits a compact histological pattern similar to most solid tumors (Fig. 7E). Our results indicated that diABZI treatment significantly altered the compactness of solid tumors (Fig. 7F–J), while TCR-T cells pre-treated with diABZI changed the compactness of tumors near blood vessels in NCG mice after tail vein injection (Fig. 7I–J).

These results indicate that the STING agonist diABZI has some antitumor effect and greatly enhances the antitumor toxicity of TCR-T cells on Mel526 tumor-bearing mice.

### STING agonist diABZI improving the antigen presentation of Mel526 tumor in NCG mice

**Fig. 8:**
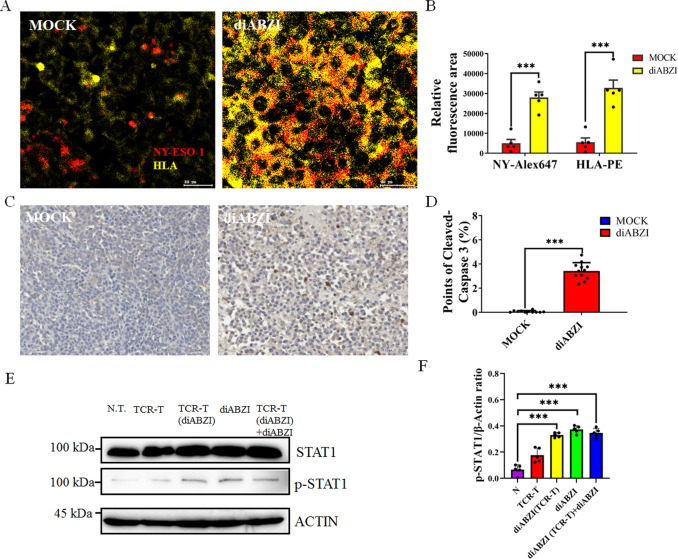
diABZI promotes the expression of tumor antigens in the tumor microenvironment. **A**, **B** Immunofluorescence imaging of NY-ESO-1 (Alex Fluor™ 647) and HLA (PE) expression with and without diABZI treatment (**A**) and analyzed by ImageJ (**B**). **C**, **D** Photomicrographs of IHC staining of cleaved caspase 3 (**C**) and analyses of caspase 3 activity response to diABZI treatment in Mel526 tumors (**D**)**. E** The phosphorylation of STAT1 increased after diABZI treatment at indicated times. **F** Analysis of phosphorylation of STAT1. ***p < 0.001.

We further detect the antigen expression in tumor microevironment, as we found that diABZI induces antigen presentation in Mel526 cells in vitro. We found that diABZI significantly enhanced the antigen presentation in tumor environment (Fig. 8A, B, Fig. S5A, B).

Our results shown that the use of diABZI alone can significantly slow down tumor volume growth and change the compactness of tumor (Fig. 7B, F). IHC results indicated that diABZI treatment increased tumor apoptosis (Fig. 8C, D). These results implied that diABZI slow down tumor growth by directly inducing tumor cell apoptosis or altering the immune environment.

It reported that phosphorylation of STAT1 increase the MHC1 antigen presentation [30]. We investigated the phosphorylation of STAT1 in cells and tumors after stimulation or treatment with STING agonist diABZI. We found that diABZI treatment significantly enhanced STAT1 phosphorylation in cells and Mel526 tumors (Fig. 8E, F, Fig. S5C, D). This indicates that the STAT1-mediated signaling pathway is involved in the promotion of tumor antigen presentation by diABZI.

We previously found that diABZI activates the STING pathway and also promotes the high expression of IL6 in vitro (Fig. 5F). We further tested the expression of IL6 in tumors treated with diABZI, and the ELISA results showed that diABZI treatment did significantly increase the release of IL6 in the tumor microenvironment (Fig. S5E). However, if we use TCR-T cells pre-treated with diABZI to be intravenous injected into the animal body, it greatly reduced the release of IL6 in the tumor microenvironment (Fig. S5E).

## Discussion

Cancer immunotherapy aims to promote the activity of CTLs in lymphoid organs and establish efficient and durable antitumor immunity. However, the antitumor activity of CTLs is usually low and unsustainable in a cancer environment. Synthetic biology suggests that genetic engineering of autologous T cells to express either chimeric antigen receptors (CARs) or affinity-enhanced T cell receptors (TCRs) that recognize known tumor target antigens may help to overcome these problems [31]. TCR-T cells have been employed in many early-stage clinical trials for melanoma [6]. However, the approaches of increasing TCR-T antitumor activity or T cell durable antitumor immunity have been receiving more attention [32].

**Fig. 9:**
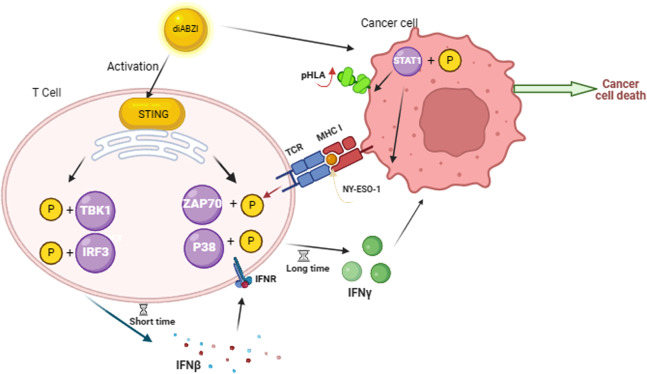
diABZI promotes T cells cytotoxicity to cancer cells by increasing INFγ production in T cells and enhancing antigen presentation in tumor cells.

It was reported that programmed cell death protein 1 (PD-1) expression on tumor-infiltrating lymphocytes (TILs) or TCR-modified T cells contributed to tumor immunotherapy [33]. This indicated that the programmed cell death pathway is important for T cell immunotherapy. Recently, STING agonists have appeared in clinical trials related to immunotherapy [34]. The STING agonists, DMXAA and cGAMP, greatly enhanced CAR-T cell persistence in the tumor microenvironment [13]. Our results found that the STING agonist diABZI is bifunctional, causing an increase in T cell activity and tumor cell antigens presentation (Fig. 9), helping the antitumor activity of CTLs and TCR-T in vitro. Moreover, we realized that diABZI transfers membranes more easily compared to cGAMP.

STING is a transmembrane protein localized to the endoplasmic reticulum and functions as an adapter protein in the cGAS (cyclic GMP-AMP synthase)-STING pathway [35], which drives activation of type I IFN and other inflammatory cytokines in the host immune response against tumors or DNA [36]. Recognition of cytoplasmic DNA by cGAS generates cGAMPs, which binding to STING induces transformational changes in STING protein, activating a downstream signaling cascade involving TBK1 and IRF3, which results in the production of type I IFNs [37]. The type I IFN profile and presence of activated CD8^+^ T cells in the tumor microenvironment have been correlated with favorable outcomes in different solid tumors [38].

Our data indicated that diABZI induced type I IFN expression after stimulating HATs for 3 h and enhanced antitumor function. The STING ability of increasing type I interferons expression revealed that the immunotherapeutic effects of many anticancer modalities depend on type I IFN signaling [39, 40]. Type I IFNs exert a variety of effects on enhancing the cytotoxic ability to secrete IFN-γ of different immune cells, including natural killer cells [19]. Our results suggest that the expression of IFN-γ in the later stage may be due to the promotion of type I interferon after diABZI stimulation at early stage (Fig. 9). Our results also showed that diABZI directly activates ZAP70 mediating signaling pathway and increase HATs IFN-γ expression after diABZI treatment in TCR-engineered T cells.

It has been reported that STING activation enhances cancer antigen presentation, contributing to the promotion of the recognition and killing of cancer cells by T cells [41]. Moreover, type I IFNs exerts their antitumor effects by enhancing the expression of MHC class I required for recognition by CD8^+^ T cells [42]. Our in vivo and in vitro data demonstrated that diABZI enhanced the cancer cell antigen NY-ESO-1 and HLA-A2 presentation and increased TCR-T activation. We also found that diABZI increased the tumor cell apoptosis in vivo and in vitro. This indicates the possibility of direct use of STING agonists antitumor therapy. However, diABZI induces the expression of IL6, a member of the pro-inflammatory cytokine family [43]. It may be helpful for T cell immunotherapy to find or develop a new molecule that does not induce the expression of IL6, and another option is to activate TCR-T and other T cells for a short period of time in vitro, and then inject them into patients. Our in vitro and in vivo data indicated that the advances in STING-targeted and TCR-T therapies provide an update on the clinical development and application of STING agonists in combination with affinity-enhanced TCR-T or other approaches.

### Supplementary information

Supplement table and figures

original file

## Data Availability

All datasets generated and analyzed during this study are presented in this published article and its Supplementary Information files. Additional data are available from the corresponding author upon reasonable request.
