# Supplementary material for: STING agonist diABZI enhances the cytotoxicity of T cell towards cancer cells
Source: Cell Death Dis. 2024 Apr 13;15(4):265. doi: 10.1038/s41419-024-06638-1 (PMC11016101; doi:10.1038/s41419-024-06638-1)
Supplement: Supplementary file 1 — Supplement table and figures [file 41419_2024_6638_MOESM1_ESM.pdf]

STING agonist diABZI enhances the cytotoxicity of T cell towards cancer cells

Ling Wang[1#], Zhaoduan Liang[2#], Yunzhuo Guo[1#], Jean de Dieu Habimana[3], Yuefei Ren[3], Obed Boadi Amissah[3], Omar Mukama[3,4], Siqi Peng[5], Xuanyan Ding[3], Linshuang Lv[3], Junyi Li[3], Min Chen[1], Zhaoming Liu[3], Rongqi Huang[3], Yinchao Zhang[6], Yi Li[5]*, Zhiyuan Li[3,7]*, Yirong Sun[3]*.

*** Correspondence:** Corresponding Author: li_yi@gibh.ac.cn(Y. Li), li_zhiyuan@gibh.ac.cn (Z. Li), sun_yirong@gibh.ac.cn(Y. Sun).

Supplementary Table 1

Gene	Primer sequences (5'->3')
GAPDH	AACAGCCTCAAGATCATCAGC（F）<br>CACCACCTTCTTGATGTCATC（R）
IL6	CAGCCCTGAGAAAGGAGACAT（F）<br>GGTTCAGGTTGTTTTCTGCCA（R）
BCL2	CGGTCTCCTGGTGCCATTAT（F）<br>GAAACGTCACCTGCCCCC（R）
IFNγ	GAAAGACAACCAGGCCATCAG（F）<br>TCATGAATGCATCCTTTTTTGC（R）
IFNβ	ACGCCGCATTGACCATCTATG（F）<br>CGGAGGTAACCTGTAAGTCTGT（R）
CXCL10	AGCAGAGGAACCTCCAGTCT（F）<br>AGGTACTCCTTGAATGCCACT（R）
BCL-xL	CCCATGCTCCGTTATCCTG（F）<br>TAAGTCGCCATCCAAGCTGC（R）
PUMA	GACCTCAACGCACAGTACG（F）<br>CCTAATTGGGCTCCATCTC（R）
BAD	GGTTCTGAGGGGAGACTGAGG（F）<br>CTCGGCTCAAACTCTGGGAT（R）
BIM	TTCTTGCAGCCACCCTGC（F）<br>CTTGCGTTTCTCAGTCCGAG（R）
NOXA	GCAAGAATGGAAGACCCTTG（F）<br>CGCCCAGTCTAATCACAGGT（R）

Supplementary Figures

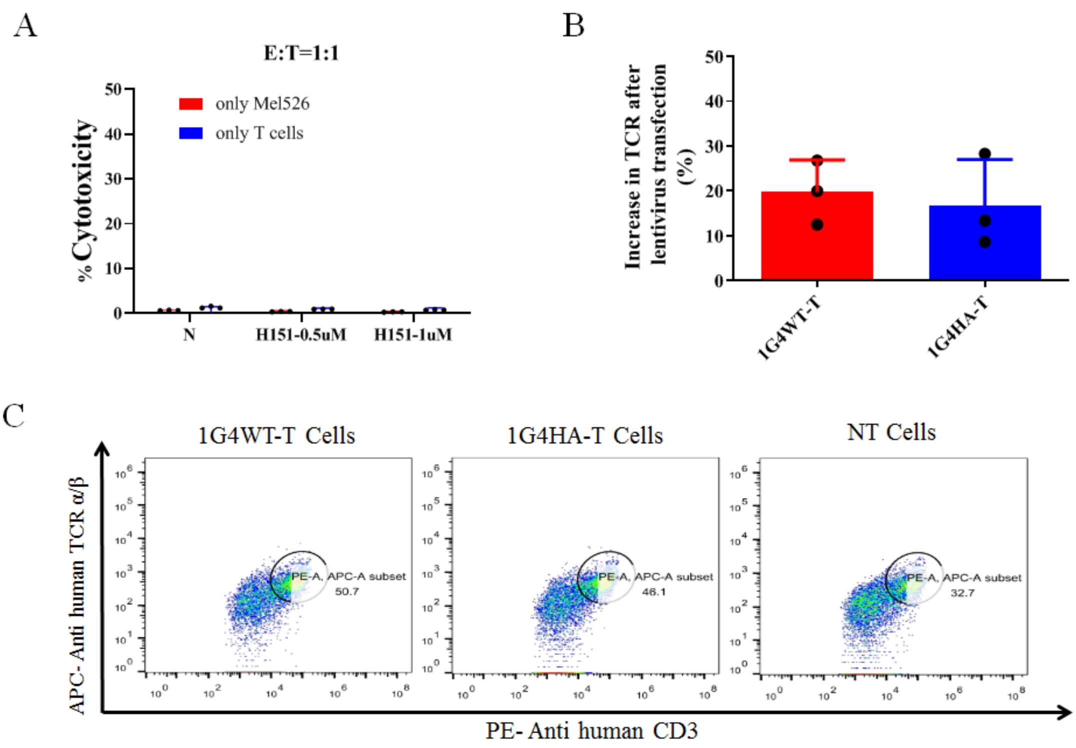

Fig. S1 TCR expression increased after 1G4 lentivirus infection. **A** Mel526 and T cells were treated with H151 for 3 hours, and then cultured with T cells at indicated ratios. **B** Increase in TCR after lentivirus transfection. **C** Flow cytometry analysis of TCR.

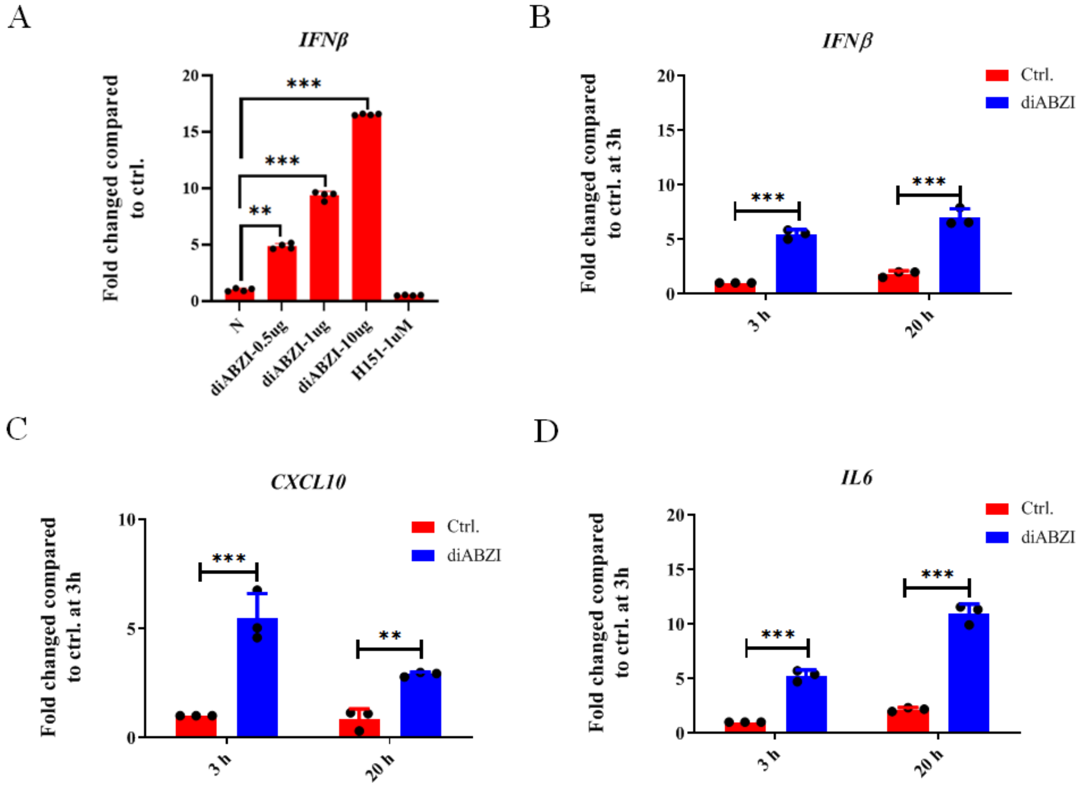

Fig. S2 Expression of *IFN-β* in HA-T (**A**) and PBL (**B**), *CXCL10* (**C**) and *IL-6* (**D**) in PBL at the time indicated after diABZI stimulation for 3 hours. **, $p < 0.01$, ***, $p < 0.001$.

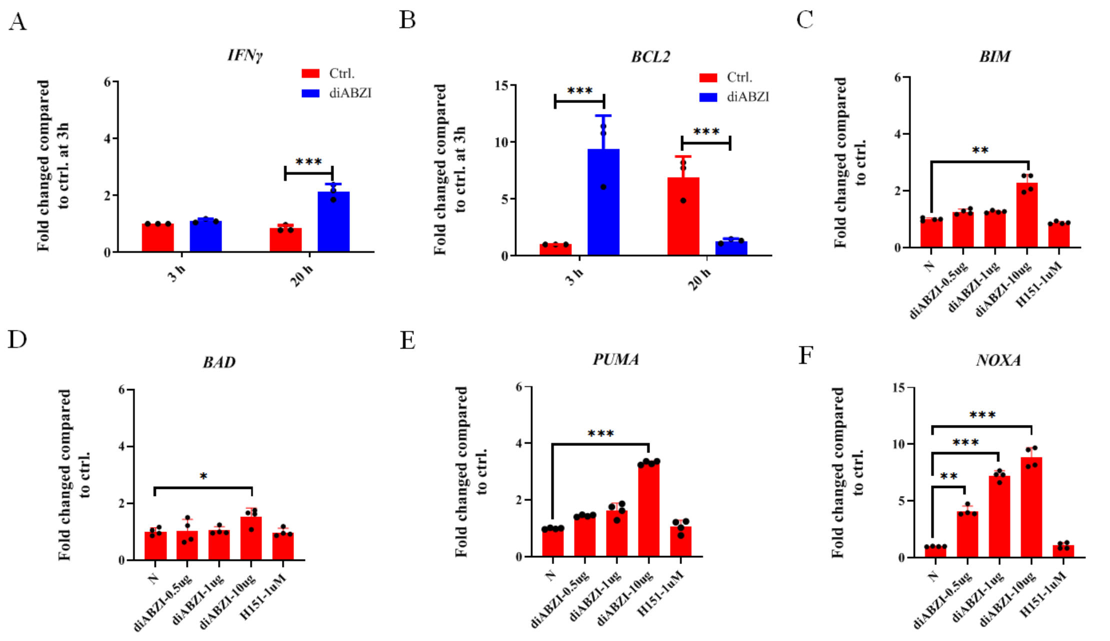

Fig. S3 IFN-γ (**A**), BCL2 (**B**) and BH3 molecules (**C-F**) expression in PBL cells at indicated time points with and without diABZI. *, $p < 0.05$, **, $p < 0.01$, ***, $p < 0.001$.

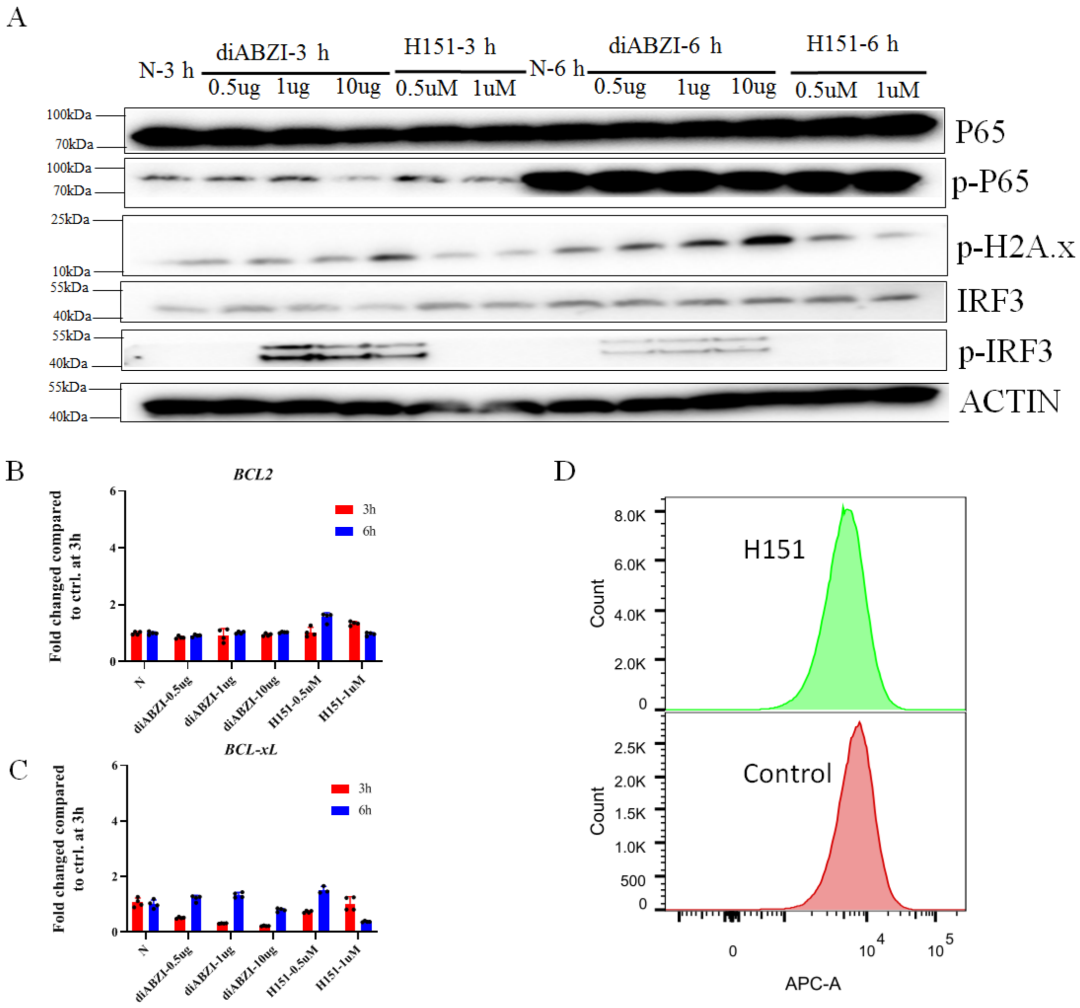

Fig. S4 A Phospholation of P65, H2A.x and IRF3 in Mel526 cells treated with STING agonist diABZI and inhibitor H151 identified by Immunoblot. **B-C** Expression of *BCL2* and *BCL-xL* in Mel526 at the time indicated after diABZI stimulation. **D** Mel526 cells were detected by FITC anti- Human NY-ESO-1 and PE anti-Human HLA-A2 after treatment with H151 for 6 hours.

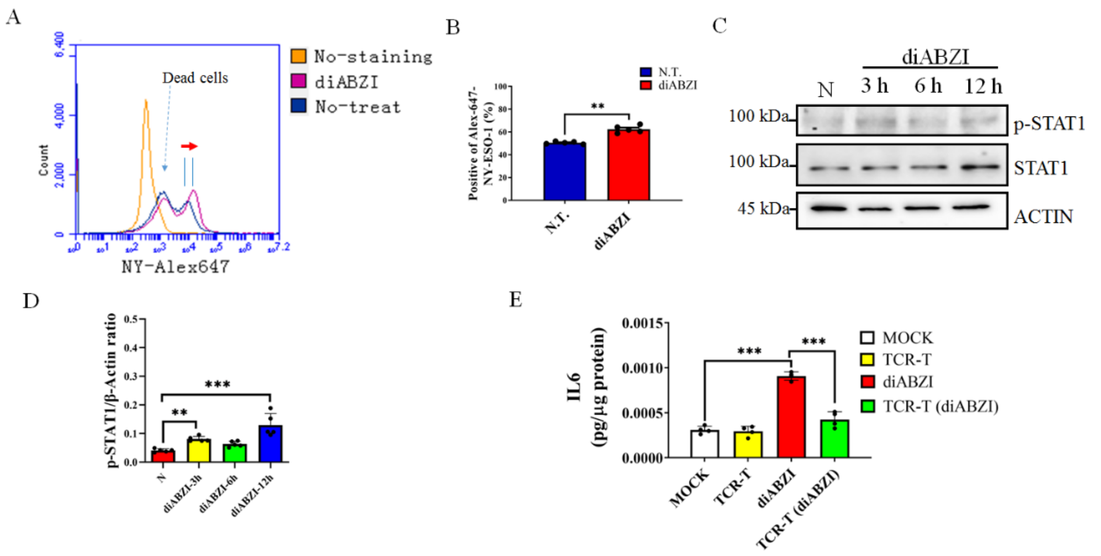

Fig. S5 A-B Mel526 cells extracted from tumors were detected by Alex Fluor™ 647 anti- Human NY-ESO-1(**A**) with and without diABZI treatment and percent of NY-ESO-1 were analyzed (**B**). N.T., non-treatment. **C-D** The phosphorylation of STAT1 increased after diABZI treatment in Mel526 cells at indicated times (h, hour). **E.** ELISA analysis of IL6 expression in tumor microenvironment with and without diABZI treatment. TCR-T (diABZI), TCR-T cells were pre-treated with 1 µg/ml 2 hours. **, $p < 0.01$, ***, $p < 0.001$.
