# Supplementary material for: STING agonist diABZI enhances the cytotoxicity of T cell towards cancer cells
Source: Cell Death Dis. 2024 Apr 13;15(4):265. doi: 10.1038/s41419-024-06638-1 (PMC11016101; doi:10.1038/s41419-024-06638-1)

Figure 1

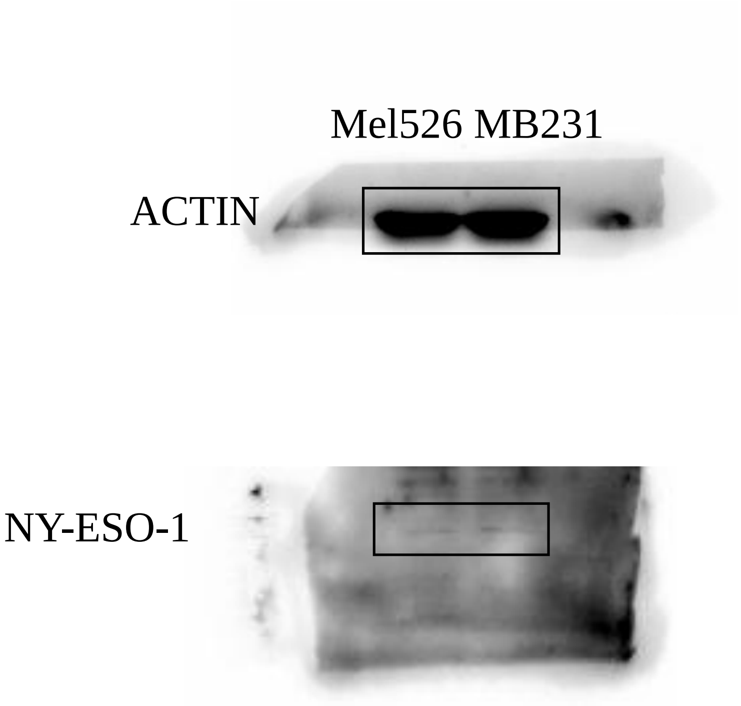

Figure 2

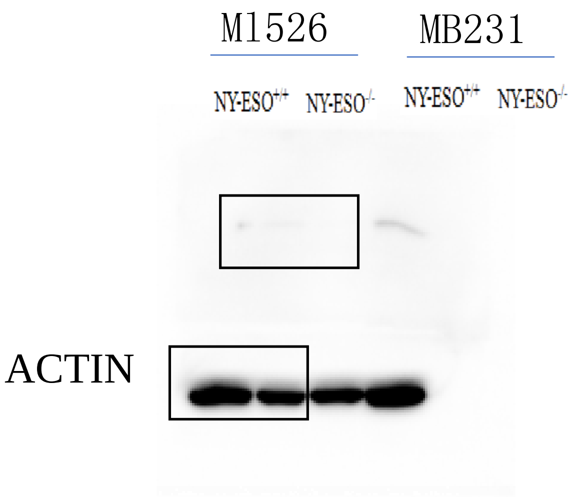

Figure 3

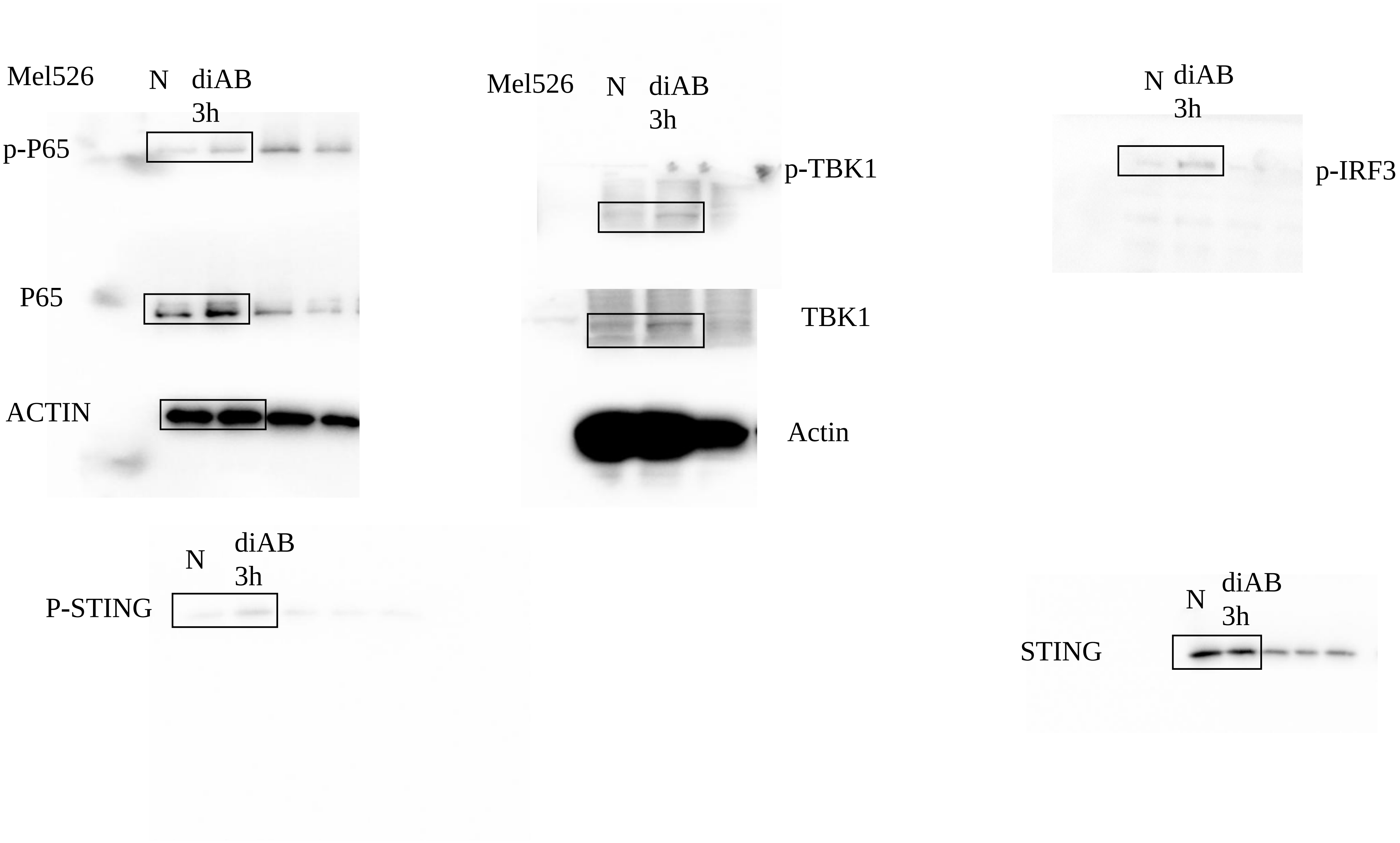
Mel526
N
diAB 3h
p-P65
P65
ACTIN
Mel526
N
diAB 3h
p-TBK1
TBK1
Actin
N
diAB 3h
p-IRF3
N
diAB 3h
P-STING
N
diAB 3h
STING

Figure 4

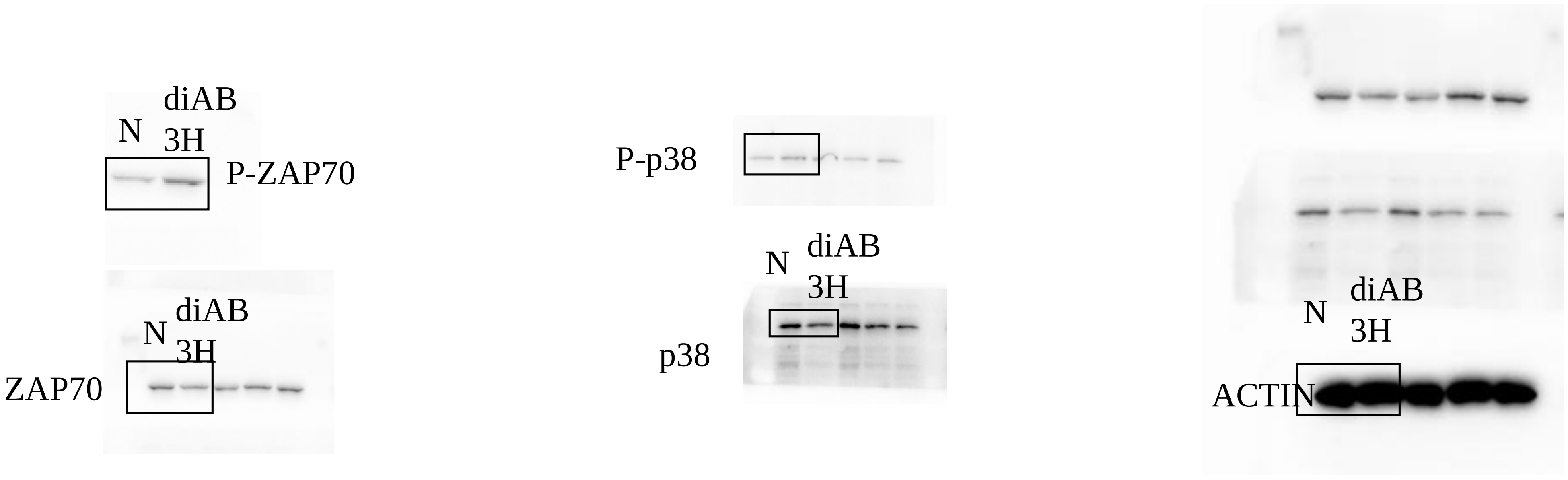
N
diAB 3H
P-ZAP70
N
diAB 3H
ZAP70
P-p38
N
diAB 3H
p38
N
diAB 3H
ACTIN

Figure 5 B

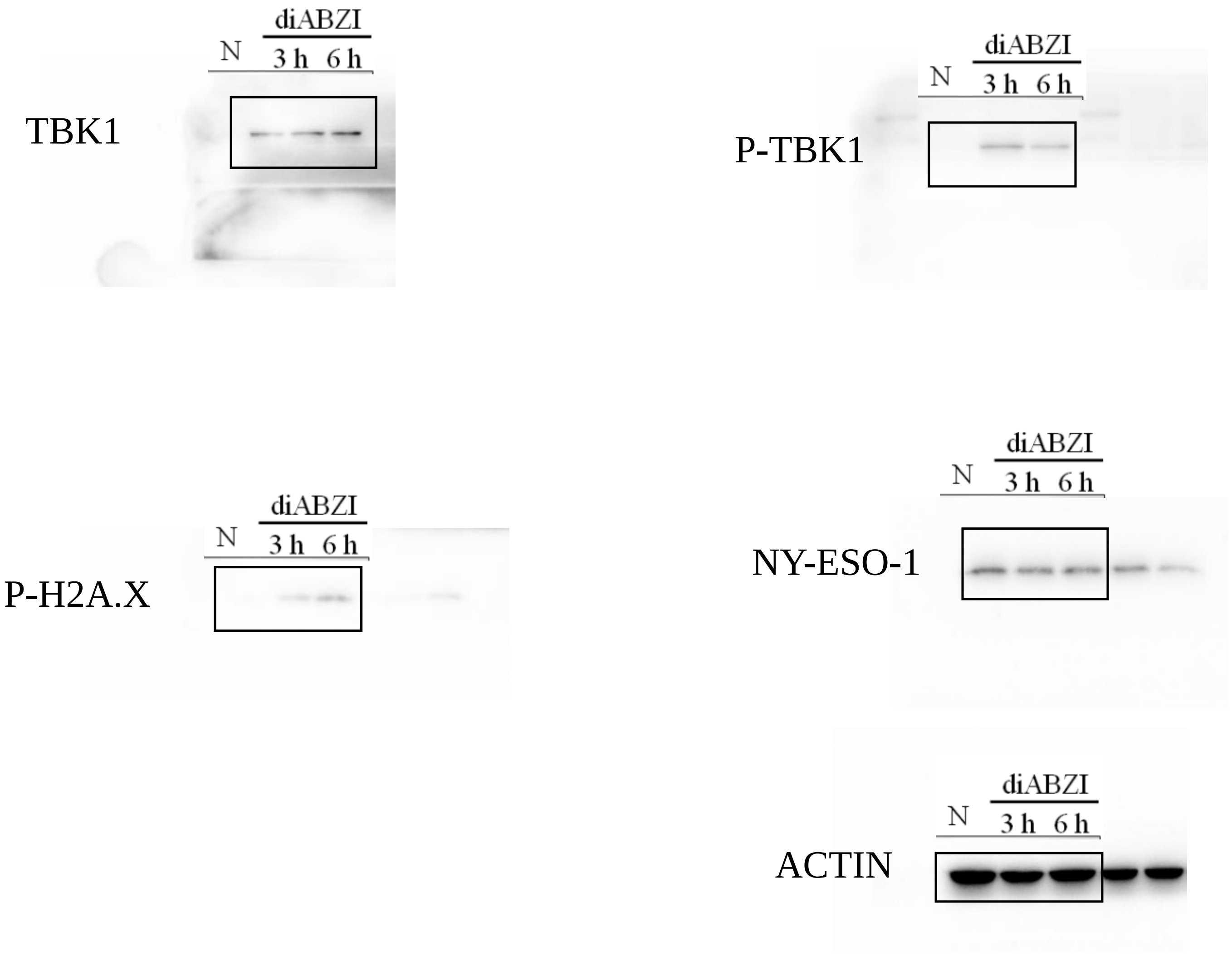

Figure 5 D

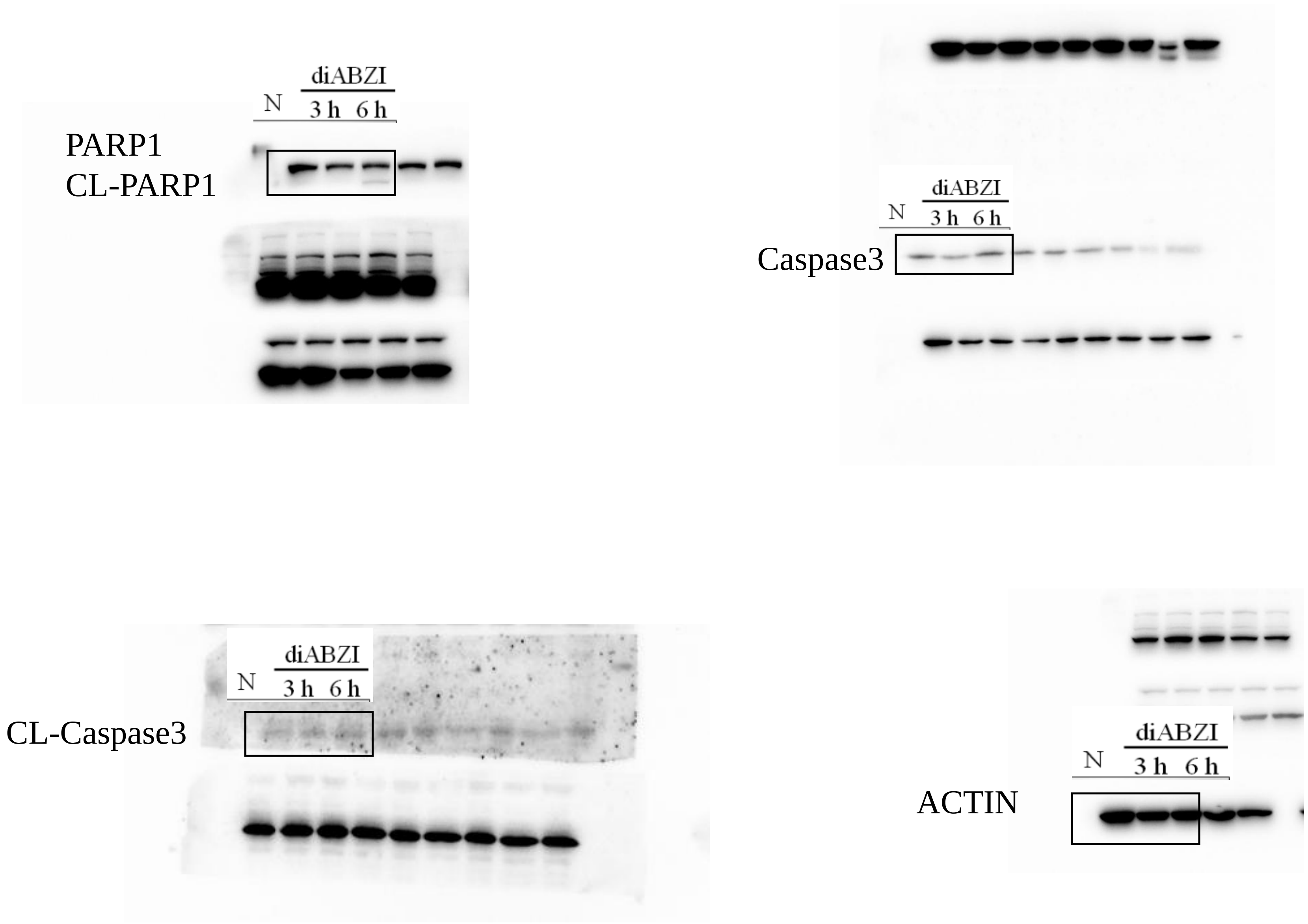
diABZI
N
3 h
6 h
PARP1
CL-PARP1
diABZI
N
3 h
6 h
Caspase3
diABZI
N
3 h
6 h
CL-Caspase3
diABZI
N
3 h
6 h
ACTIN

Figure 7C

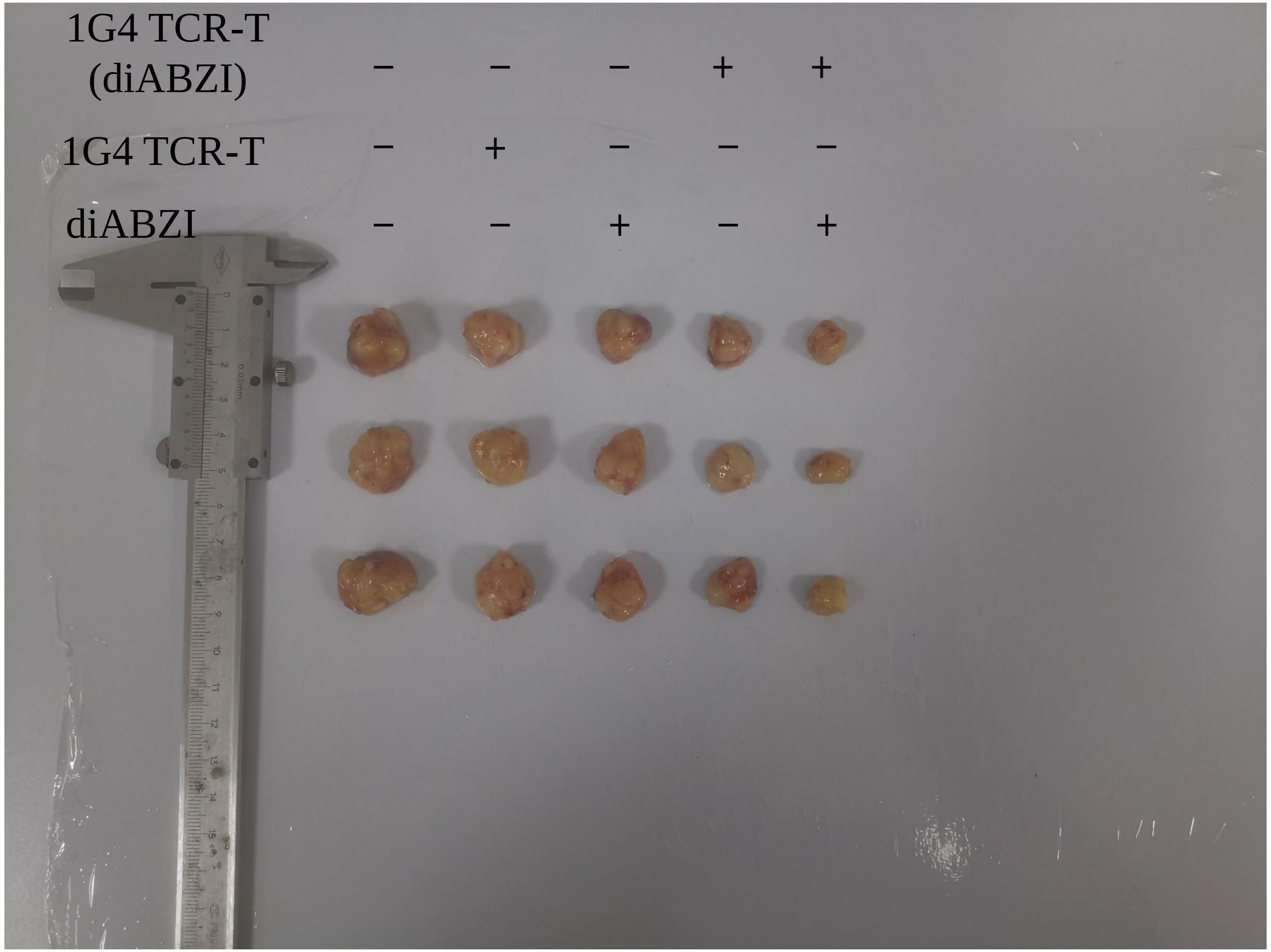

Figure 8E

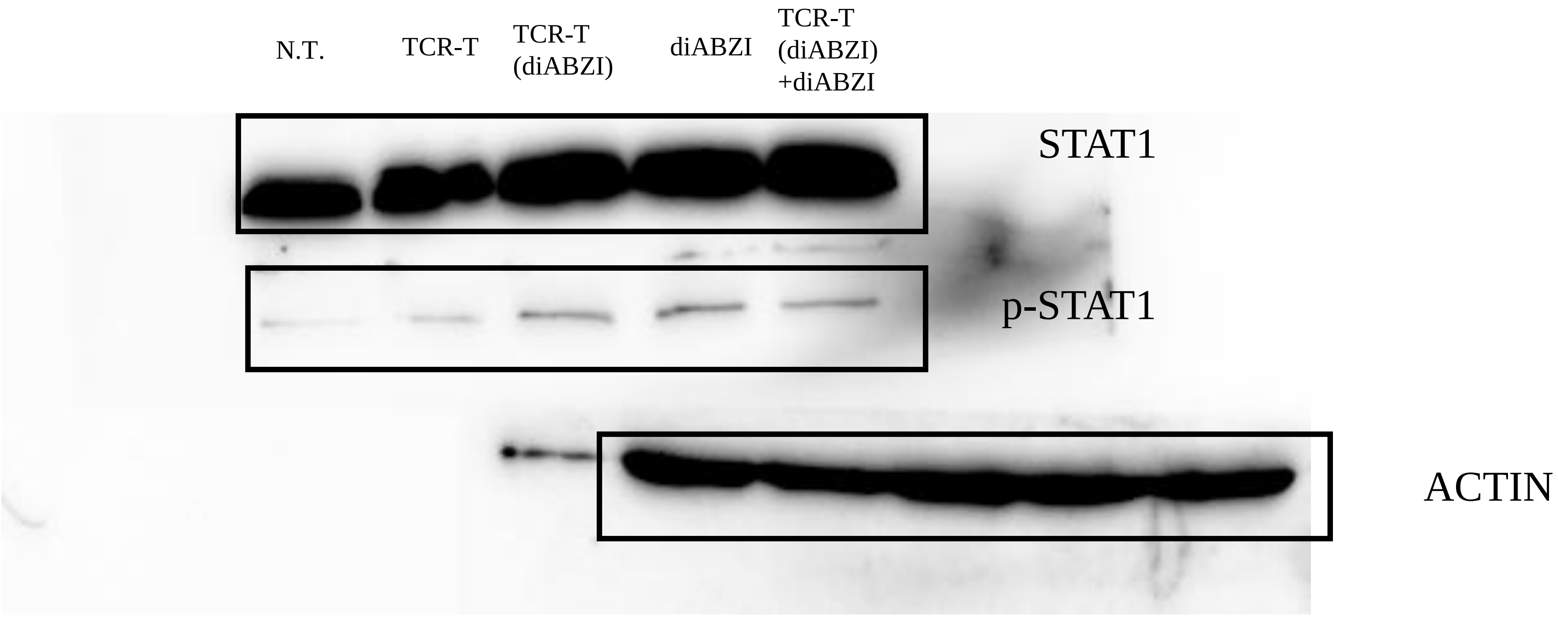
N.T.
TCR-T
TCR-T (diABZI)
diABZI
TCR-T (diABZI) +diABZI
STAT1
p-STAT1
ACTIN

Figure Supplementary 4 A（1）

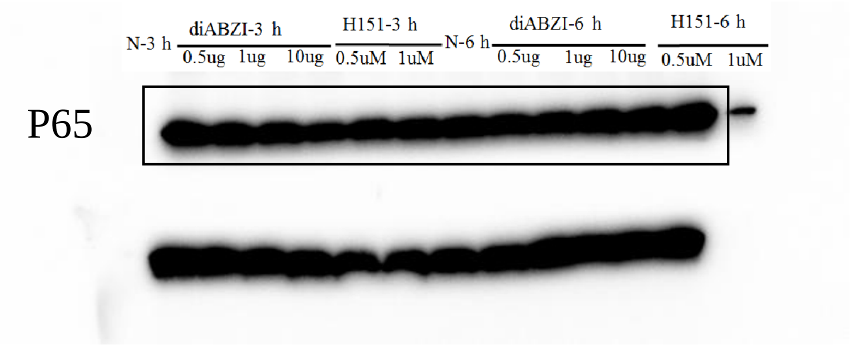

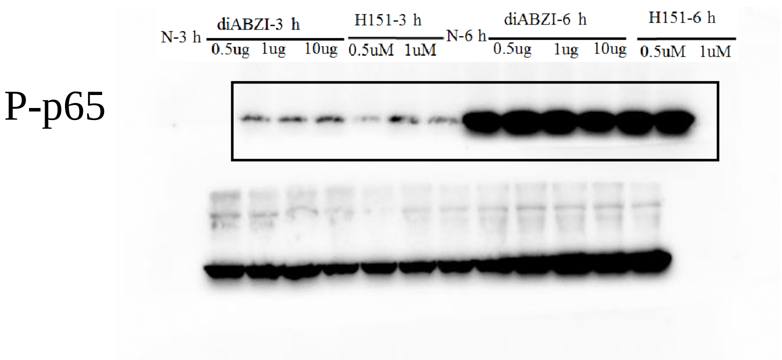

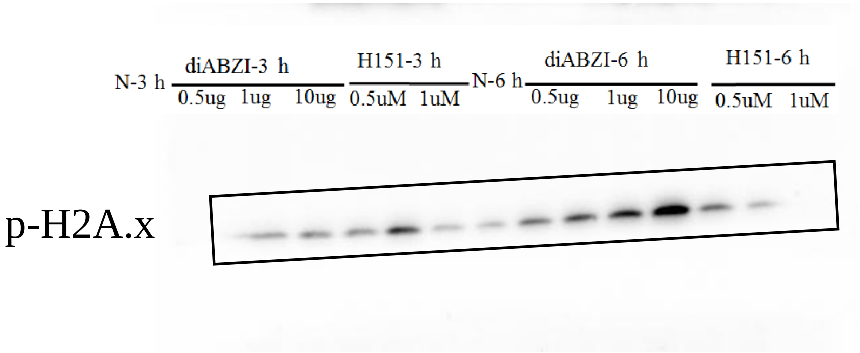

Figure Supplementary 4 A（2）

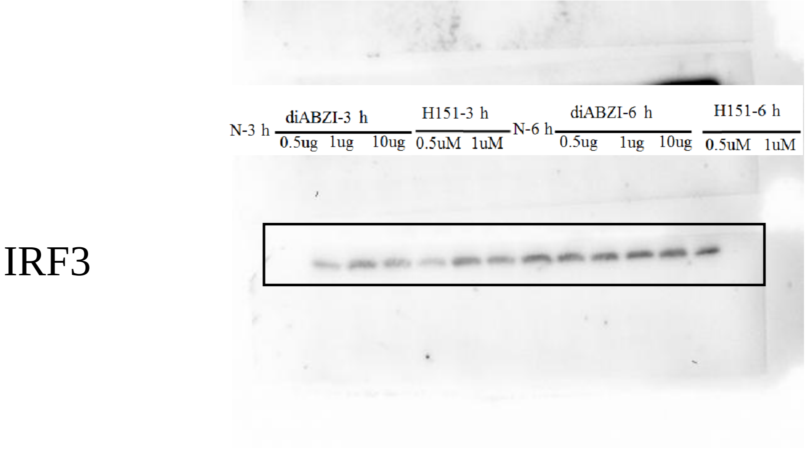

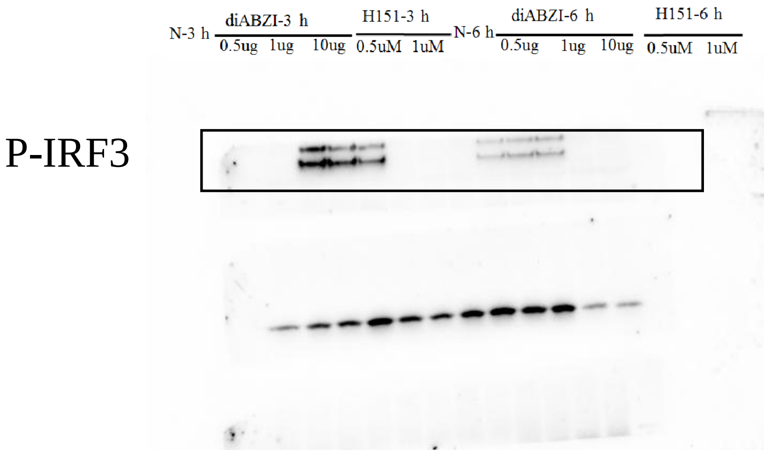

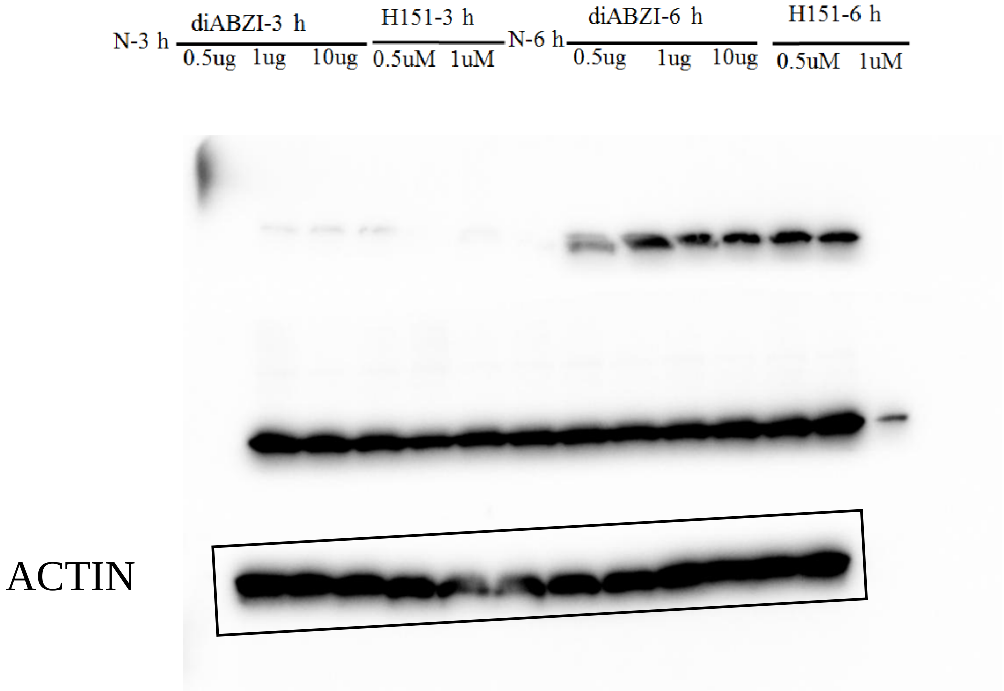

Figure Supplementary 5 C

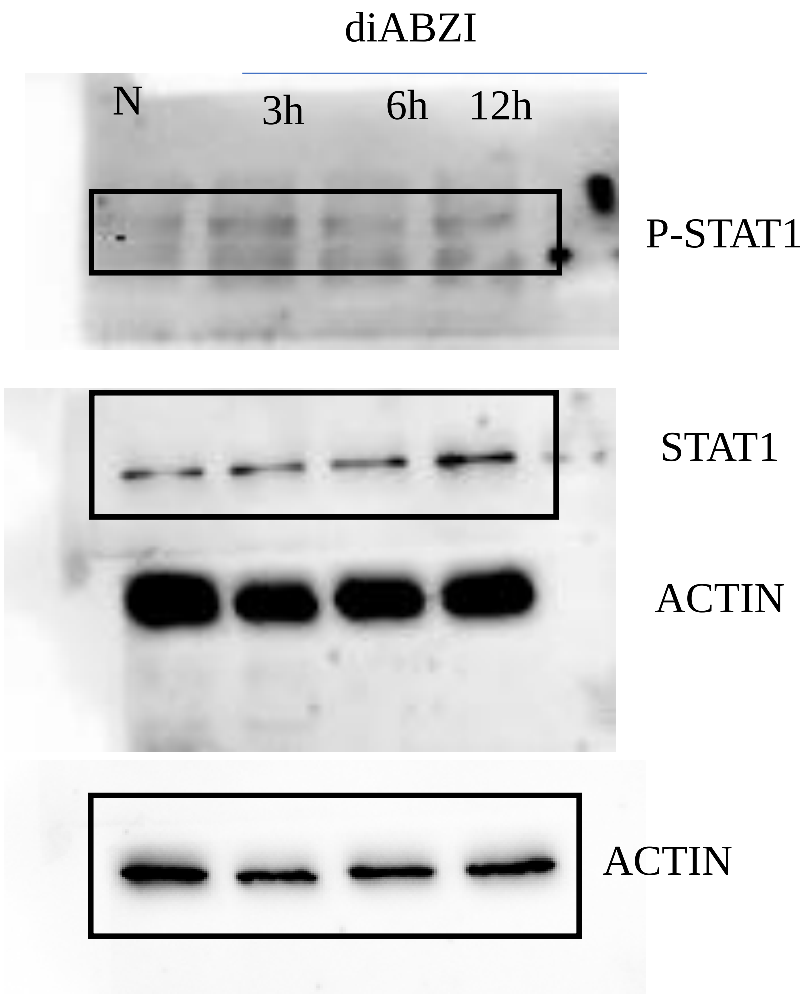

Supplement: Supplementary file 2 — original file [file 41419_2024_6638_MOESM2_ESM.pdf]
